# Exploring the engagement behaviours of Smile4life practitioners: lessons from an evaluation of the national oral health improvement programme for people experiencing homelessness in Scotland

**DOI:** 10.3389/froh.2023.1289348

**Published:** 2024-01-04

**Authors:** Laura Beaton, Andrea Rodriguez, Gerry Humphris, Isobel Anderson, Ruth Freeman

**Affiliations:** ^1^ School of Dentistry, University of Dundee, Dundee, United Kingdom; ^2^ School of Medicine, University of St Andrews, St Andrews, United Kingdom; ^3^ Faculty of Social Sciences, University of Stirling, Stirling, United Kingdom

**Keywords:** oral health, homeless persons, qualitative, COM-B model, behaviour

## Abstract

**Introduction:**

Smile4life is Scotland's national oral health improvement programme for people experiencing homelessness, aimed at reducing oral health inequalities experienced by this population. This study forms part of an evaluation of how the Smile4life intervention was being implemented within Scottish NHS Boards. The aim was to investigate the influence of the Smile4life intervention upon the engagement behaviours of Smile4life practitioners.

**Methods:**

Focus groups were conducted with Smile4life practitioners, to provide an insight into how the Smile4life intervention affected their skills, attitudes and experiences while interacting with people experiencing homelessness and their services providers. A purposive sample of oral health practitioners, including dental health support workers, oral health promoters/educators, and oral health improvement coordinators working in three NHS Boards were invited to take part. One focus group was conducted in each of the three NHS Boards. The focus groups were audio-recorded and transcribed. The COM-B model of behaviour was used as a framework for analysis.

**Results:**

Eleven Smile4life practitioners took part in the focus groups. All had first-hand experience of working with the Smile4life intervention. The average focus group length was 67 min. Working on the Smile4life intervention provided the Smile4life practitioners with: (i) the capability (physical and psychological), (ii) the opportunity (to establish methods of communication and relationships with service providers and service users) and (iii) the motivation to engage with Third Sector homelessness services and service users, by reflecting upon their positive and negative experiences delivering the intervention. Enablers and barriers to this engagement were identified according to each of the COM-B categories. Enablers included: practitioners' sense of responsibility, reflecting on positive past experiences and success stories with service users. Barriers included: lack of resources, negative past experiences and poor relationships between Smile4life practitioners and Third Sector staff.

**Conclusion:**

The Smile4life programme promoted capability, provided opportunities and increased motivation in those practitioners who cross disciplinary boundaries to implement the Smile4life intervention, which can be conceptualised as “boundary spanning”. Practitioners who were found to be boundary spanners often had a positive mindset and proactive attitude towards the creation of strategies to overcome the challenges of implementation by bridging the gaps between the NHS and the Third Sector, and between oral health and homelessness, operating across differing fields to achieve their aims.

## 1 Introduction

People experiencing homelessness often experience social exclusion, as well as poorer oral health and oral health-related quality of life and a higher prevalence of dental decay than the general population (1, 2). Smile4life is Scotland's oral health improvement programme for people experiencing homelessness. It was developed in 2007, with the intention of addressing the oral health needs of the homeless population of Scotland and reducing the health inequalities experienced by this group. An intervention and supporting resources for practitioners was launched in 2012 (3). The aim of the Smile4life intervention was to build the capacity of NHS and Third Sector^1^ staff to address the oral health needs of patients or service users experiencing homelessness, which could include providing information and resources, support, or facilitating access to dental care. Smile4life remains the only health programme in the country focusing on the links between oral health/health and homelessness. The intervention is intended to be delivered by the oral health teams from the NHS boards in Scotland through engagement with health and social care sectors and the provision of training for service users and practitioners. Smile4life adopted the European Typology of Homelessness, acknowledging anyone who was roofless or houseless (residing in insecure or inadequate accommodation) as experiencing homelessness (4). Therefore, service users receiving the Smile4life intervention are a diverse range of people experiencing homelessness, including people in temporary accommodation, rough sleepers visiting soup kitchens or homeless drop-ins, and others in more long-term accommodation.

A process evaluation of the Smile4life intervention was conducted in the 18 months following the launch of the intervention. The aim of this evaluation was to evaluate the implementation of the intervention in the NHS Boards. Interviews with NHS practitioners from across Scotland revealed variation in the adoption and implementation across the NHS Boards (5). The Boards that more readily adopted Smile4life were those with perceived knowledge and skills to effectively communicate and form partnerships with different stakeholders, but other Boards faced barriers to implementing the intervention. This suggested that there was a need for a more in-depth exploration of how Smile4life was being implemented, in order to fully understand the factors that influenced practitioners and organizations, and explore behaviours associated with the delivery of the intervention.

Prior to this study, a participant observation study took place with Smile4life practitioners in three Scottish NHS Boards (1). The purpose was to observe their delivery and implementation of the Smile4life intervention in community and primary care settings. The participant observation study suggested that for the Smile4life intervention to be implemented effectively, there must be a strong triadic working alliance between the Smile4life practitioner, Third Sector staff and service users. The findings suggested that when Smile4life is being delivered successfully, the Smile4life practitioners appeared to be adept and interested in creating chances to interact with service users and Third Sector staff. The observation study also explored differences in how the intervention was being delivered, which had been initially recognised during the earlier process evaluation (1, 5). For example, one NHS Board opted to provide clinical services for people experiencing homelessness, while in the other areas the practitioners focused on providing information and support.

Questions still remained regarding the effect of the Smile4life intervention upon the practitioners' behaviours, and whether it was possible that the intervention itself acted as a stimulus to promote their abilities to engage with clients and Third Sector staff. In order to examine this proposition, it was necessary to return to the Smile4life practitioners and find out their thoughts and opinions about the intervention, and ask them to reflect on their experiences, including how it assisted them in their working practices with homeless service users. Therefore, the aim of this study was to investigate the influence of the Smile4life intervention upon the engagement behaviours of Smile4life practitioners as they worked towards tackling the health inequalities of people experiencing homelessness.

## 2 Method

### 2.1 Sample and recruitment

A purposive sample of oral health practitioners working in three NHS Boards were invited to take part. These boards had participated in the earlier observation study. Participants were invited if they worked firsthand with Smile4life delivery, either providing training for staff or delivering oral health advice directly to people experiencing homelessness. The group of practitioners targeted were: Dental Health Support Workers, Oral Health Promoters, Oral Health Educators, or Oral Health Improvement Coordinators.

Recruitment emails were sent out to Oral Health Managers from the three NHS Boards, who were asked to disseminate to their teams. Participants from the earlier observation study were contacted directly. Practitioners that were interested in taking part were told to contact the research team, who then sent them the participant information sheet and consent form to read. After one week, the research team contacted the practitioners, all of whom had agreed to take part, to arrange a date for the focus group.

### 2.2 Data collection

Focus group discussions were chosen as the data collection method, to provide an insight into how the Smile4life intervention affected the skills, attitudes, and experiences of the practitioners as they delivered and implemented Smile4life. Focus groups also allowed participants, as end-users of the Smile4life intervention, to voice their experiences and opinions about the best ways of delivering the intervention. Seeking feedback from practitioners as part of the evaluation process ensured that the aims of the research team align with the needs of the end-users and allowed the practitioners to be active collaborators in evaluating the intervention (6).

Focus group sessions were conducted in small groups (7–10). Morgan and Krueger recommended that focus groups should be non-judgmental, meaningful and friendly, and Bloor et al. noted that there can be benefits to using pre-existing groups (8, 11). Many of the participants already knew the facilitator (LB), either from the observation stage of data collection or from earlier research that had been conducted as part of an evaluation into Smile4life implementation (2, 5). This ensured that there was a pre-existing rapport between the participants and the facilitator before the focus groups took place. Since the practitioners knew each other and the facilitator, and were part of a pre-existing national group, this allowed them to feel comfortable, for an easy flow of shared experiences to be described and ensured that there was a more true-to-life discussion.

For the focus group discussions, the questions were designed to meet Krueger and Casey's recommendations for good quality, e.g., conversational, short, clear and easy to understand, gradually moving from the general to the specific (12). The questions asked participants about: who they were; their experiences interacting with service providers and service users; the skills needed to be a Smile4life practitioner; the risks involved in Smile4life work; perceptions of homelessness and what helped when interacting with people experiencing homelessness. The full list of questions is presented in Supplementary File S1. Prompts were used to clarify the question or to suggest possible answers if the participant was unsure of how to respond and were often based on the observations made during the previous stage of research. Focus groups were conducted in person. All three focus groups requested that the discussion take place in their place of work. Therefore, a meeting room or office space, where the group would not be interrupted, was the setting for the focus group discussions. Each focus group was audio-recorded, with the recordings being transcribed by LB. No reimbursement was provided to participants for taking part.

### 2.3 Data analysis

The transcripts were analyzed using framework analysis. Framework analysis is a method of qualitative data analysis suitable for research that has “*specific questions… or a priori issues*” to consider and can be used to “*describe and interpret what is happening in a particular setting*” (13). For this qualitative exploration, the COM-B model was used as the framework. COM-B is part of the Behaviour Change Wheel (BCW), a framework of behaviour change interventions, which was used to underpin the overall evaluation of the Smile4life intervention, of which this study forms one part (12). The BCW is concerned with interventions for behaviour change, specifically developing and/or improving existing interventions. As Smile4life is an intervention aimed at assisting dental health professionals' facilitatory actions when interacting with people experiencing homelessness, it was hoped that by investigating Smile4life using the BCW, recommendations could be made to inform this and future interventions to improve practitioners' interactions and behaviours to promote oral health within the homeless population.

The COM-B model sits at the center of the BCW and focuses on the sources of Behaviour (B): Capability (C), Opportunity (O), and Motivation (M). The use of the COM-B model allows for the identification of factors that influence the occurrence of a behavior (14).

Srivastava and Thomson identified five stages to the data analysis when using framework analysis: (i) familiarization; (ii) identifying a thematic framework; (iii) indexing; (iv) charting; (v) mapping and interpretation (13). From the initial analysis using the three components from the COM-B model, it became apparent that COM-B was an appropriate framework with which to continue analysis—this meant that the themes and codes used to analyze the data were pre-selected based on the elements of the COM-B model. A second read-through was conducted to index any and all data that fitted the COM-B model (e.g., examples of practitioners' capability, opportunity and motivation), and anything else that arose from the data. This process was repeated for each transcript—the indexing from each was then collected together, to establish common themes. This was then entered into a framework matrix, essentially a chart summarizing the data based on the themes that emerged for each category (Supplementary File S2). As part of the final analysis, attention was also paid to identification of boundary spanning activities and roles. This analysis was conducted by hand by LB in the first instance, with regular discussions between LB and RF to review and refine themes.

### 2.4 Ethical considerations

Ethical approval was applied for and granted by the University Research Ethics Council at the University of Dundee (UREC 15098). Consent forms had to be read and signed before the focus group could take place. All data were anonymised before analysis. No ethical issues arose during data collection or reporting.

## 3 Results

In total, eleven Smile4life practitioners from three NHS Boards agreed to participate. All were female. While these practitioners had a variety of job titles, all had experience working with the Smile4life intervention. The sample represented the key people involved in the Smile4life programme in their respective Boards. Table 1 illustrates the number of practitioners that took part in each focus group, as well as the diversity of job roles represented in each group. The focus group discussions lasted between 57 and 74 min, with an average length of 67 min.

**Table 1:** Focus group participants.

NHS Board	Number of participants	Gender of participants	Job roles of participants
1	5	All female	Dental health support worker
			Oral health educator
			Oral health coordinator
2	2	All female	Oral health promoter
3	4	All female	Dental health support worker
			Oral health training officer
			Health improvement practitioner

### 3.1 Capability

The transcripts were analyzed to determine whether or not the Smile4life intervention had affected the practitioners’ engagement behaviours with regard to their psychological and physical capabilities.

Overall, in each of the three NHS Boards, implementing Smile4life facilitated the practitioners' physical capability since it ensured that they had the physical resources (e.g., toothbrush packs provided by their NHS Board, copies of the Smile4life Guide for Trainers) to achieve their behavioral aims. In general, Smile4life also ensured that they had the psychological capability for a consistent service provision. However, elements of both physical and psychological capability also acted as barriers to consistent service delivery of Smile4life, e.g., perception of risk (Table 2).

**Table 2:** Capability category—engagement enablers and barriers.

Category	Subcategory	Results	
		Enablers	Barriers
Capability	Physical	Availability of physical resources including human resources	–^a^
	Psychological	Knowledge of Smile4life and of homelessness	Perceptions of risk
		Tailoring the intervention	
		The use of incentives as a tool for engagement	
		Availability of skills needed to do the job (e.g., life experience, communication skills, empathy)	Negative perceptions or expectations of homelessness
		Resilience	

^a^ - = this subcategory was not found.

Availability emerged as the most significant dimension of being physically capable of delivering Smile4life. The practitioners spoke of being physically available, having the appropriate job role to deliver oral health messages, and the availability of people to provide the service associated with the delivery of the programme. In Board 3, for instance, one of the practitioners was in a post that was created solely for the purposes of delivering the Smile4life intervention. The Smile4life intervention gave the staff the means and ensured that staff were physically available (i.e., there was an availability of resources, including staffing), and provided opportunities for increasing psychological knowledge and skills about how to engage with homelessness services. It was, therefore, possible to conceptualize the characteristics of “capable” Smile4life practitioners as having the physical resource capability (e.g., their physical availability) and psychosocial capability (e.g., being dependable/reliable). Comments from the Smile4life practitioners illustrated that they felt that being physically available and psychologically dependable was particularly valuable to Third Sector services and homeless service users who were used to a fast turn-around of outside visitors such as the Smile4life practitioners:

*“…the (other) services come in and they’re not consistent—they don't turn up when they say they’re going to turn up, or the person leaves and a new person starts, or the funding is taken away… but now they know we’re going to turn up every week, it's fine. I think that helps, and having the same person, not swapping people round.” (Participant1_Board2)*

#### 3.1.1 Physical capability: availability of resources

The availability of physical resources included having toothbrushes packs, a mobile dental clinic, and having the necessary staff to deliver the Smile4life intervention. Toothbrush packs were frequently used as an incentive to help facilitate engagement. In one participating NHS Board, these NHS-regulation packs were supplemented by free samples of Oral-B and other branded products. The practitioners agreed that the offer of a toothbrush pack facilitated discussions with service users about their oral health, as well as providing the tools required to maintain good oral health. In one NHS Board the offer of free samples was extended to Third Sector staff as a way of developing and strengthening relationships:

*“It's a tool for engagement. I always make sure the staff have got theirs as well… if you’re helping them, they’re more willing to help you.” (Participant1_Board2)*

In Board 1, the Smile4life practitioners were physically capable of engaging with and addressing the treatment needs of people experiencing homelessness because they had access to a mobile dental unit (MDU). The MDU was a physical resource not available in every Board. It acted as the primary setting for the consistent and regular delivery of Smile4life in Board 1 and provided the Smile4life practitioners with the physical space to deliver the Smile4life intervention, to speak to service users and to offer dental treatment. It should be noted, however, that the focus within the MDU was providing dental treatment, not oral health promotion and in this respect was perceived as a potential barrier to the implementation of the Smile4life programme. One practitioner from Board 1 commented during the focus group that they were unsure if the MDU could be considered as an appropriate delivery resource for the Smile4life intervention. Therefore, despite having the physical capability to provide dental treatment and oral health promotion, the MDU appeared to act as a barrier, preventing the Smile4life practitioners in this Board from engaging with homeless services or service users outside of the confines of the MDU.

#### 3.1.2 Psychological capability: knowledge

Practitioners' knowledge emerged as an element of their psychological capability. The practitioners' knowledge was composed of their oral health knowledge and their personal knowledge and experience of working with those in the homelessness sector together with people experiencing homelessness. The following quote is illustrative and implies that the Smile4life intervention facilitated additional learning experiences for the Smile4life practitioners beyond oral health.

*“If you’ve done some sort of oral health education, which we had to do for our job, and then training sessions, we attend poverty awareness sessions, health inequality sessions, so we’ve got a good background on health inequalities, and I think if you’ve got that, it helps, it can help you understand why these people are there in the first place.” (Participant5_Board1)*

While some practitioners had prior experience of working with people that were experiencing homelessness, and therefore, some knowledge about homelessness issues, the majority were not familiar with this population before working on Smile4life. They spoke of “*having their eyes opened*” to the realities of homelessness while delivering Smile4life and how the implementation of Smile4life had increased their awareness and knowledge of homelessness issues. Smile4life had psychologically prepared them for engaging with Third Sector homelessness services and service users.

When asked about their thoughts on homelessness before they began working on Smile4life, the majority of Smile4life practitioners reported that they were initially surprised by the variety in age, background and circumstances of people experiencing homelessness, for example, that people who were experiencing homelessness could be families or older people, not just young, single people or people with a history of alcohol and/or drug use. A common theme that emerged during the focus group discussions was initial surprise that some people experiencing homelessness had come from “*good backgrounds*” or were “*well educated*” yet had ended up homeless. Being involved with the Smile4life intervention had expanded practitioners' views of people facing homelessness, beyond the common negative stereotypes. A better understanding of homelessness aided them when engaging with Third Sector homelessness services and service users.

Practitioners stressed the importance of tailoring the way they delivered Smile4life in order to encourage engagement with and from the Third Sector staff and service users:

*“You kind of tailor to the best time… it's just trying to make it bespoke to what fits”. (Participant2_Board2)*

The importance of tailoring was included as part of the Smile4life training and implementation guidance, suggesting that the practitioners were putting their knowledge of how to deliver Smile4life into practice, in order to increase engagement. Tailoring was also a way of interpreting the needs of the Third Sector service and service users, to facilitate engagement, whereby the Smile4life practitioners were working across sectors (NHS and Third Sector) to provide their oral health services.

#### 3.1.3 Psychological capability: skills

Another key factor of psychological capability was having the psychosocial skills or abilities required to carry out a task. Common themes relating to the required skills emerged from all three focus groups. The practitioners suggested a set of important skills to deliver the intervention: effective communication, specifically listening skills; empathy; conflict resolution; sincerity; approachability; confidence; flexibility; and an ability to be non-judgmental. One Smile4life practitioner summed this up more simply:

*“You have to be able to be a human being”. (Participant1_Board3)*

Another common skill was stated as life experience:

*“If you’re older and bit more mature, I suppose, you have life skills”. (Participant1_Board1)*

*“I think it's because we’re old and we have life experience!” (Participant1_Board2)*

When the above skills existed for the Smile4life practitioners it seemed to indicate that they had the most appropriate approach to work on Smile4life, which in turn gave them the chance to engage with people within the homelessness sector. This notion that working on Smile4life was a job that would suit particular people was supported by this statement from one Smile4life practitioner during the focus groups:

*“You have to employ the right person to do the job, they have to want to do it”. (Participant1_Board2)*

This suggested that, although Smile4life appeared to increase practitioners' capability via training and increased knowledge and skills, it still required a certain type of individual who could use the intervention to combine the training with their own life experiences to promote their engagement with homeless service users and Third Sector services.

#### 3.1.4 Psychological capability: risk and resilience

One potential psychological barrier that emerged from the observation study was the notion that working on Smile4life could be perceived as risky, with service users observed as being unpredictable and disruptive. If practitioners felt they were at risk, this could potentially pose a threat to their psychosocial capability to engage with service users. Therefore, a question was posed during the focus groups to find out if the Smile4life practitioners themselves believed their job was risky. Initially, all Smile4life practitioners said “*No*”, denying that they felt afraid or at risk while working on Smile4life. However, when asked to elaborate on this, some Smile4life practitioners revealed situations where they had been frightened. For instance, one Smile4life practitioner spoke about her own experience with a service user who had bitten her. Others discussed the methods they used to minimize risk or de-escalate situations should there be any early signs of a potentially risky scenario:

*“If something kicked off, I know that I could run up the street to get away from it” (Participant1_Board1)*

*“If they are becoming agitated or swearing, I’ll bring it down immediately… you can do things that you know will de-escalate it” (Participant1_Board2)*

Not only were the Smile4life practitioners psychologically capable of overcoming such potential risks, whether by denying there was a risk or devising strategies to de-escalate situations, physical steps were also taken by the NHS Boards to protect the Smile4life practitioners:

*“In the best possible way, our management are very risk averse! With the intention that they have to keep the staff as safe as possible”. (Participant2_Board3)*

Two out of the three participating NHS Boards revealed that they use services such as Guardian24 and Reliance Protect, essentially an emergency service connected to a Smile4life practitioner's ID badge:

*“There's a pin alarm on here, there's an alert button, people can call in and decide what the situation is.” (Participant1_Board3)*

These devices did not remove the possibility of a risky or dangerous situation arising, but they provided a safeguard and may have minimized the sense of risk felt by Smile4life practitioners, which, in turn, would increase their capability to engage with Third Sector homelessness services and service users. In addition, Smile4life practitioners reported carrying their own personal alarms or alarms and radios provided by the service.

#### 3.1.5 Overall capability: summary

It emerged that working on the Smile4life intervention provided the Smile4life practitioners with the capability—both physical and psychological—to engage with Third Sector homelessness services and service users. Because of Smile4life, the practitioners had the physical capability to engage with service users about their oral health. In Board 1, in particular, Smile4life had resulted in the use of a MDU in order to reach service users. However, this also acted as a barrier to further engagement with services, as the MDU was seen as being sufficient, and no further attempts at engagement with other services were made. For those working in Board 1, while it may be surmised that whilst the MDU improved capability in its physical form, the apparent lack of psychological capability reduced the effect of the Smile4life programme to increase engagement with service users and Third Sector services. In all three Boards, Smile4life enabled practitioners to improve their knowledge of homelessness issues, and the skills needed to engage with services and service users, including the importance of tailoring the intervention and the use of incentives to facilitate engagement. In addition, the Smile4life intervention enabled engagement as it challenged practitioners' perceptions about homelessness, increasing their understanding of this population and hence their working behaviours.

### 3.2 Opportunity

In order for Smile4life practitioners to engage with Third Sector services and service users as part of delivering Smile4life, they had to have opportunities that allowed them to do so—did the Smile4life intervention provide the opportunities they needed or were there social influences that influenced the opportunities to enable their engagement behaviours? From the focus group discussions, it became apparent that, for the most part, Smile4life practitioners did have these opportunities. For example, the Smile4life intervention facilitated physical access to service users, but there were also several barriers that prevented engagement with Third Sector services and service users, such as dental anxiety from the staff or service users (Table 3).

**Table 3:** Opportunity category—engagement enablers and barriers.

Category	Subcategory	Results	
		Enablers	Barriers
Opportunity	Physical	Access to service users	-^a^
	social	Strong relationship with Third Sector	Dental anxiety
		Finding key people	
		Support from NHS Board	Difficulties engaging with the third sector

^a^ - = this subcategory was not found.

#### 3.2.1 Opportunity: physical and social opportunities with third sector

##### 3.2.1.1 Access to service users and relationships with third sector

The most noteworthy way that the Smile4life intervention provided practitioners with the opportunity to engage was by giving the practitioners a reason to access service users. This access allowed them to speak directly to service users about their oral health, give advice or signpost to relevant services. This access, however, was mediated by the social influence of the Third Sector staff, who often acted as both enablers and barriers to this opportunity for engagement with service users. In this instance, the two subcategories of opportunity overlapped, with both physical access and social opportunities impacting upon the engagement behaviours of the Smile4life practitioners.

When Smile4life practitioners could not interact with the Third Sector staff, either because the service was not interested or because of a breakdown in communication, it was difficult for them to implement Smile4life as they could not reach the service users—Board 1's Smile4life practitioners, for instance, recalled particularly negative experiences when interacting with their local Third Sector organisations:

*“(The services) weren't that keen. They didn't get back to you about it”. (Participant4_Board1)*

When asked to expand on possible reasons for this lack of engagement, Board 1's Smile4life practitioners suggested that it was due to these Third Sector services not having the time, or having limited staff, to deal with Smile4life, or having other priorities for their service and their service users, which did not include oral health:

*“It's not a case of not being interested, it's more a cause of them just saying “we don't have time”, “we have other priorities”, “we’ve got enough to do””. (Participant5_Board1)*

The practitioners in Board 1 seemed satisfied with the service provision in the MDU, but all agreed that earlier attempts to engage with services had not been positively received. They described the different ways they had attempted to engage—offering training, providing drop-in sessions—but felt that the services only wanted them to signpost or give out toothbrush packs. It appeared that they had stopped trying to do more than this. Nevertheless, it may be possible to speculate that after so many knock-backs from Third Sector services, Board 1's Smile4life practitioners themselves had become disinterested, did not have time, and had other priorities. Indeed, one practitioner explained that their main priority was an oral health programme for people in care homes.

It seemed that, for Board 1, with regard to opportunities to engage, Smile4life did not always act as an enabling factor in the initial stage of accessing Third Sector services. However, with perseverance, practitioners in other Boards were successful. Board 3, for example, also reported difficulties engaging with Third Sector services initially, but found that they had to make their own opportunities, both social and physical, either at a frontline level or at a strategic level, interpreting the needs of the services, and the most appropriate way to establish a relationship:

*“It's just getting ourselves established on that agenda”. (Participant3_Board3)*

Nonetheless, factors unrelated to Smile4life appeared to increase engagement and this included the prevalence of homelessness within a particular area. In Board 2, for example, the Smile4life practitioners that took part in the focus group were responsible for two different geographical areas within the Board. They spoke of the variety in the way they were welcomed and received by Third Sector services. In one area, where there was a higher homeless population and a faster turnaround in hostels, the staff were more helpful; in another part of the Board's geographical area, where there were fewer homeless people, and service users often remain in one accommodation for a number of years, the staff were a barrier to engagement with service users. The following quotes are illustrative:

*“In other places they’ll do a knock-up in a hostel… there's a poster up the week before, there's a leaflet drop the night before underneath their doors and at room check the staff will say “(The OHP's) down the stairs, do you have any problems?”” (Participant1_Board2)*

*“Staff I feel are my barrier here… there are ones where I feel I’m hitting my head against a brick wall… when you go in the staff are kind of “oh well, no one wants to see you today” rather than let the clients make that decision. And they’re not as forthcoming to knock them out their bed”. (Participant2_Board2)*

##### 3.2.1.2 Finding key people

Smile4life practitioners from Boards 2 and 3 acknowledged that Smile4life had facilitated opportunities for engagement with key people within the local authority or Third Sector who were supportive of Smile4life and were in a position to help the Smile4life practitioners access Third Sector services and service users, as commented upon by practitioners working in Boards 2 and 3:

*“She (a Health and Homelessness lead for a local authority) was a great help, she was another link, she's obviously very senior, very supportive… she coordinated the whole thing for us, which was wonderful”. (Participant3_Board3)*

*“The right individual to make it happen. You need to find the one that can invite you in, the one that can smooth the waters, the one that can give you what you want”. (Participant1_Board2)*

The oral health managers or coordinators, some of whom took part in the focus groups, also found key people who could provide opportunities to engage with services and service users by accessing Third Sector managers or local authority leads via meetings and discussions at a strategic level. This allowed Smile4life to be discussed with audiences at a higher level and ensured that the Smile4life practitioners were able to access services that they might not have been able to before:

*“My senior manager sits at more of a strategic level with the movers and shakers of the service providers… there's still a lot of people that don't know about Smile4life… you can see it start to filter through”. (Participant2_Board3)*

##### 3.2.1.3 Dental anxiety

Aside from the need to improve relationships with some Third Sector services, Smile4life, or more generally the combination of oral health and homelessness, was responsible for a range of social factors that could negatively affect opportunities to implement Smile4life. The first of these raised was dental anxiety. This was commented upon by practitioners in two of the NHS Boards, but for different reasons.

In one, the dental anxiety of Third Sector staff was cited as a potential reason for poor engagement from one service to the extent that when the Smile4life practitioner visited this service, one member of staff would try to avoid her. Because of this, the Smile4life practitioner had been provided with an opportunity to engage and began to talk to this member of staff about her oral health and help her overcome her dental fears. The Smile4life practitioner believed that it helped her to engage with the Third Sector staff.

In the other Board, the Smile4life practitioner cited a lack of available resources about dental anxiety. She believed that having such resources would provide an additional opportunity for engagement with fearful service users, since it was one of the main barriers service users faced with regard to addressing their oral health needs. The Smile4life practitioner felt that these resources would provide her with more opportunities to engage with service users.

#### 3.2.2 Opportunity: social opportunities with NHS boards

Smile4life practitioners were asked directly if they would work in oral health and homelessness if Smile4life did not exist. The practitioners stated that while some work in this area would have taken place, it would not be to the extent now that the Smile4life programme existed:

*“It's one of the priority groups that the Community Dental have to see, so I think we would still see them as patients and signpost them, but I don't think you’d have much interaction”. (Participant3_Board1)*

*“No, because there was nothing happening before… I don't think anything would be happening”. (Participant2_Board3)*

Therefore, it would seem that Smile4life was an enabling factor providing practitioners with opportunities to engage with homelessness. However, Smile4life practitioners in Board 2 stated that they would have been tackling oral health and homelessness anyway, with or without Smile4life or policies from the Scottish Government:

*“It was something that I was interested in anyway… I was bored at work and I thought “nobody's doing this”… at the time our manager would back you and say “have a bash, see how it goes, see what happens””. (Participant1_Board2)*

This quote suggests that the Smile4life intervention provided an opportunity at the Board level to allow their oral health practitioners to engage with the homelessness sector. Therefore, this example from Board 2 suggested that support from the Board—in this instance, the practitioner's manager—also provided practitioners with an improved opportunity to engage with services and service users, as well as validating work already being undertaken.

#### 3.2.3 Overall opportunity: summary

For Smile4life practitioners to engage with services and service users, they needed both physical and social opportunities to do so. These were often interconnected: to gain physical access to service users, Smile4life practitioners had to first build strong relationships with the Third Sector, which they accessed by finding key people to help them, or by addressing the wider issues of dental anxiety. The role of the NHS Board to facilitate engagement was also noted.

### 3.3 Motivation

Motivation can be divided into reflective and automatic motivation. Reflective motivation refers to instances where decision-making is based on rational thought, i.e., an individual reflects on a decision, taking into consideration facts and experience. Automatic motivation is where decisions are made based on how a person—or in this context, how a Smile4life practitioner—feels, an emotional response (14). Both opportunity and capability can influence motivation (12). Both reflective and automatic motivation will now be discussed in relation to the focus group discussions (Table 4), as well as how the previously discussed examples of capability and opportunity acted to influence this motivation.

**Table 4:** Motivation category—engagement enablers and barriers.

Category	Subcategory	Results	
		Enablers	Barriers
Motivation	Automatic	Positivity in the face of negative experiences	–^a^
	Reflective	Success stories from service users	Negative past experiences
		Reflecting on past experience	
		Sense of responsibility	

^a^ - = this subcategory was not found.

#### 3.3.1 Reflective motivation

##### 3.3.1.1 Success stories

A common example of reflective motivation for Smile4life practitioners was success stories or positive feedback from service users. These served to buoy Smile4life practitioner's motivation to continue delivering Smile4life and to engage with services and service users. The practitioners reported being pleased or satisfied when they thought about the emotional responses from service users:

*“One of the service users I spoke to… she had lost both her dentures, she’d lapsed with her addiction, she was back in recovery, she said: “I really want to get my smile back, I’m really glad you’re here today’ and I just thought, that really shows you the need for it”. (Participant3_Board3)*

*“The best one for me, I was up in town shopping on a Saturday and someone came up to me and said, “I’m smiling because of you””. (Participant1_Board3)*

##### 3.3.1.2 Sense of responsibility

Policies regarding oral health and homelessness, such as the Dental Action Plan and the National Oral Health Improvement Strategy for Priority Groups, provided an opportunity for Smile4life practitioners to engage with services and service users but they also motivated Smile4life practitioners (15, 16). As with success stories, the fact that policies existed gave Smile4life an inherent worth and significance, and validated the work undertaken by some of the oral health practitioners already working with people experiencing homelessness. When Smile4life practitioners were asked if they would still approach the oral health of people experiencing homelessness without Smile4life, some Smile4life practitioners noted that they would have to do something because of the existing policies in place that dictate what NHS Boards should do with regard to homelessness. While this motivated Smile4life practitioners, it is evidently a reflective, not automatic, decision for most, and is perhaps considered more of a task that is completed because it has to be, in accordance with policy, not because it was a subject that they were particularly passionate about or had an emotional response to or as one practitioner stated:

*“Not every employee would want to do that, if it's taking up your own evenings”. (Participant2_Board2)*

As discussed in the capability section above, some Smile4life practitioners tailored their delivery of Smile4life to the needs of specific services and used their knowledge of Smile4life to forge their own opportunities to engage and strengthen relationships with Third Sector services' staff and service users. Their engagement behaviours also demonstrated the reflective motivation of the Smile4life practitioners, as it pointed to Smile4life practitioners having reflected on what works and what does not work, and then making a plan to overcome any barriers to engagement. Furthermore, Smile4life practitioners needed to be motivated to tailor their approach and remain flexible so that they could meet the needs of a service by often going above and beyond their normal job role and in this respect cross the disciplinary boundaries between oral health and homelessness and between themselves and their colleagues in the Third Sector.

The willingness to engage and work within the Third Sector indicated that a Smile4life practitioner was especially dedicated to their work. This characteristic was also seen in instances where the Smile4life practitioner felt responsible and hence motivated and duty-bound to carry out their Smile4life work. In the following example the Smile4life practitioner describes a sense of duty and motivation to the service who are providing her with the opportunity to access their service users, as well as to the Third Sector staff themselves.

*“(If) you don't turn up, it's a waste of their time isn't it? Because they’ve got lots of things to do on their agenda. So, if you’re not turning up and they’ve got people who are in pain…” (Participant1_Board2)*

##### 3.3.1.3 Reflecting on past experiences

Reflective decision making could sometimes demotivate Smile4life practitioners as they reflected on their negative past experiences or on their struggles to connect and engage with services. This indicated the extent to which opportunity and motivation were interconnected. Barriers to social opportunities for engagement, for instance, could lead to low motivation and had the potential to affect the Smile4life practitioners’ psychosocial capability.

*“It is a hard slog”. (Participant3_Board3)*

*“We did get involved with one unit, but the uptake with the clients was dreadful, so we haven't done much since”. (Participant4_Board1)*

This last quote suggested that current Smile4life actions in Board 1 were being demotivated by past negative experiences to the extent that there had been no subsequent attempts to engage with Third Sector services. The belief about potential consequences appeared throughout the focus group discussions and appeared to demotivate some Smile4life practitioners more readily than others:

*“We can always go in and hand in toothbrushes and toothpaste, put up posters… but what are they doing with it?… I don't know if all our stuff is sitting in a store room somewhere gathering dust”. (Participant2_Board1)*

As discussed in the capability section, certain Smile4life practitioners had the psychological capability to overcome potentially risky situations that occasionally arose when working on Smile4life and engaging with homeless service users. This ability also affected a Smile4life practitioner's motivation, because to be motivated about Smile4life, to continue working in an environment, or with a population, which may be risky, the practitioner must overcome negative past experiences. For the Smile4life practitioners who took part in the focus group discussion, it seemed as if they reflected on their experiences and concluded that although there may be risks involved, they did not feel at risk, nor would they let the potential for risk prevent them from continuing to deliver Smile4life. In this sense they had the psychological capability to not let this potential concern impact their job.

*“I’ve never come across a situation where I’ve thought I’m not safe here”. (Participant1_Board3)*

Such comments as, “*(I’ve) never felt awkward… it's not going to stop me going back*”. ensured that the potential risks involved with Smile4life did not detract from a Smile4life practitioner's motivation to carry out their job—indeed some Smile4life practitioners did not perceive these situations as risky. However, there was an acknowledgement in one Board that this attitude in the face of documented risks was perhaps a symptom of Smile4life practitioners' naivety or complacency about their own safety.

#### 3.3.2 Automatic motivation

The capability of Smile4life practitioners to not take offence at others' negative responses to them and towards Smile4life also aided in their motivation to deliver the intervention. This positivity, as illustrated in the following quotes, indicated a more automatic form of motivation, where Smile4life practitioners' own feelings are taken into consideration.

*“I think (rejection of Smile4life) it's not necessary at you, so you shouldn't take that on board… I’m never offended if someone says “nope, not interested””. (Participant1_Board2)*

*“I’m not compliant, I’m not going to be rolled over by them… you’re trying to do your job, but you don't want to be made a fool of”. (Participant1_Board2)*

Automatic motivation can also be seen in instances where Smile4life practitioners demonstrate a genuine interest in homelessness or discussing empathizing with people experiencing homelessness that they have interacted with during their Smile4life work. This was also apparent when Smile4life practitioners discussed their perceptions and awareness of homelessness—some were already familiar with or interested in homelessness, but others had no idea what to expect when they first started working with this population. For Smile4life practitioners who did have negative preconceptions of homelessness, they confessed that they were initially apprehensive, which negatively influenced their motivation to deliver Smile4life. The Smile4life practitioners soon realized that their preconceptions did not match the reality and that their experiences delivering Smile4life had given them a better understanding of the homelessness experience.

“*I think I was very much quite ashamed of myself for my preconceived ideas about what homelessness was, and it's actually totally nothing like what you think it is”. (Participant3_Board3)*

*“I was scared… just because I’d never worked with—that sounds horrible—those kind of people… but it was alright once you got talking to them. They’re just normal people”. (Participant3_Board1)*

#### 3.3.3 Overall motivation: summary

In summary, Smile4life practitioners were predominantly motivated to engage with Third Sector services and service users by reflecting upon their positive past experiences delivering Smile4life. These reflections were often positive. For example, practitioners in each of the three focus groups discussed success stories—instances where they had helped or motivated a service user to improve their oral health. However, some Smile4life practitioners dwelled on previous negative experiences (e.g., rejection from a Third Sector organisation) and let this demotivate them from attempting to engage further with that service. Therefore, while the Smile4life intervention appeared to provide the ingredients for engagement, when previous attempts at engagement had not been successful, or concerns about the risks involved had not been resolved, the Smile4life intervention was unable to motivate those practitioners to engage with homelessness services and service users.

## 4 Discussion

If we consider the behaviour element of the COM-B model to be engaging with the Third Sector and service users, it is apparent that the Smile4life intervention and programme provided the majority of Smile4life practitioners with the capability, opportunity and motivation to increase their engagement behaviours, but effective communication skills, an open-minded approach and a consistent attitude and desire to overcome barriers seemed to be pivotal. It may be proposed that the Smile4life programme promoted capability, provided opportunities and increased motivation in those practitioners who cross disciplinary boundaries. Williams conceptualized this working practice as the ability to “*boundary span*” (17).

The COM-B model of behaviour appeared to be a good fit for the focus group data. The developing themes first noted in the observational study were apparent, providing a sense of credibility to the findings of the focus group study. It became apparent that there was considerable overlap between opportunity and capability, particularly with regard to physical capability and physical opportunities provided by the Smile4life intervention. Moreover, with regard to opportunities for engagement, the social influences from the Third Sector directly influenced, positively and/or negatively physical opportunities. Furthermore, in agreement with the COM-B model, both capability and opportunity were found to influence motivation, particularly regarding the practitioners' experiences of interacting with the Third Sector.

### 4.1 Boundary spanners

A key factor influencing the behaviours of Smile4life practitioners was how the Smile4life programme influenced the engagement and relationship between the NHS practitioners and the Third Sector. In Boards which recognised the importance of oral health care within homelessness, Smile4life was successfully delivered, with the establishment of relationships and regular interactions with services and service users. In many respects, it may be proposed that the Smile4life intervention permitted the practitioners, through their improved capability, opportunity and motivation, to engage with a number of different groups within the homelessness sector and in this sense to fit the category of boundary spanners as described by Williams (17).

Williams explained this behaviour in terms of boundary spanning, an essential element to increase ongoing collaboration with regard to public policy, originally focusing on poverty (17). While early research into collaboration focused on participating organisations, research on boundary spanning looked at the role of the individual in the collaboration process. This is an important consideration, as Williams noted that “*feedback from diverse individuals engaged in collaborative working consistently championed the pivotal role of key individuals in shaping outcomes*” (17). During the analysis of the focus groups, Smile4life practitioners sought out key people within the Public or Third Sector who could provide them with opportunities for engagement. This finding suggested that the practitioners spanned within and beyond their organisations to find an individual who would support the delivery and implementation of Smile4life. Without the Smile4life intervention and policy documents such as the Dental Action Plan, the practitioners would not have worked across disciplines to ensure the programme's delivery (15).

To be a boundary spanner, the practitioner would, therefore, be exposed to a wide range of opinions, working environments and cultures as reflected in the Smile4life practitioners who took part in this study. They were knowledgeable regarding the practices and culture of homelessness organisations, as well as the homelessness and housing policies of their local authorities. Their past work experience or awareness of health and homelessness issues, together with opportunities for engagement, appeared to be beneficial for Smile4life practitioners whilst boundary spanning (17).

Williams noted four significant roles of a boundary spanner: the reticulist, the entrepreneur; the interpreter; and the organizer (17, 18). The reticulist aspect of boundary spanning is responsible for networking and communication and managing differing policies between the multiple agencies involved in a task. The entrepreneur is focused on innovation and creativity in the face of policies; part of this creativity and entrepreneurship involves “*risk-taking and opportunism*”, both characteristics which could be attributed to Smile4life practitioners (18). The interpreter is responsible for establishing and maintaining relationships via communication skills such as empathy and listening. The last component of boundary spanning is the organizer—the responsibility to plan and coordinate the collaborative process, taking into consideration the transfer of information between collaborative partners. Both of these components were also identified among the Smile4Life practitioners.

The four roles of Williams' boundary spanning theory explain the engagement behaviours promoted by the Smile4life intervention, which include the particular characteristics of some of these Smile4life practitioners (17, 18). The findings suggested that Smile4life practitioners who can use the Smile4life programme to facilitate multidisciplinary working are those who represent elements of all four boundary spanning roles, but particularly the entrepreneur and the interpreter. It may be proposed that the intervention promoted their capability, opportunity and motivation to engage and take on the roles of the entrepreneur and interpreter. In order to do this, the Smile4life practitioners must be creative in seizing all available opportunities. It appeared they did so by tailoring their delivery to the needs of individual services, using incentives to facilitate engagement, and often being opportunistic in approaching service users, sometimes taking risks to do so. Forging opportunities, they worked hard at maintaining relationships and engaging with Third Sector services and service users. In conclusion, it seemed that Smile4life gave practitioners the capability, opportunity and motivation to do so, and to boundary span.

Williams acknowledged that as well as these four components of boundary spanning, practitioners who are boundary spanners must also have the necessary knowledge, which has already been established through the COM-B analysis of the focus group discussions (18). Moreover, Smile4life practitioners had the necessary knowledge and the psychological capability to deliver Smile4life and engage with Third Sector services and service users. Williams also noted that “*the most effective boundary spanner exhibits certain types of personality or personal attributes*”, suggesting that extroverted personalities are particularly well suited to boundary spanning, by being positive, upbeat and outgoing, as well as working hard and being committed to the job (18). Comments from Smile4life practitioners in the focus group discussions reinforced the view that not everyone was necessarily suited to working on Smile4life—it takes the “*right kind of person*”. The right sort of person being someone who is motivated and capable of using the Smile4life intervention to promote their engagement behaviours to interact effectively with Third Sector services and service users. In Boards where Smile4life practitioners had a strong engagement pattern with Third Sector services, it was clear that the Smile4life practitioners all had characteristics in common, namely: an outgoing nature, good communication skills, and a certain fearlessness to approach people. Moreover, they were able to discuss Smile4life in potentially risky situations. Indeed, these are some of the characteristics that the practitioners themselves identified as being necessary for people who work on Smile4life. In the focus groups, this emerged as an element of psychological capability, indicating that the Smile4life practitioners were the right people to do the job.

### 4.2 Implications

The findings from the focus groups form part of a larger evaluation of the Smile4life intervention (1, 19, 20). They have demonstrated how Smile4life is delivered within NHS Boards and highlighted areas where improvements, or changes, may be made for future Smile4life work. It is hoped that by understanding ways in which Smile4life delivery could be improved, the intervention will reach a wider range of people experiencing homelessness, and as such, help to meet this population's oral health needs. These recommendations may also be transferable to other health interventions for homeless populations, or for interventions aimed at people with multiple exclusion, such as people in prison or Gypsy/Traveller communities.

By investigating the practitioner factors that influence Smile4life, this research has unpicked the complexity of the implementation of the Smile4life intervention and contributed to our understanding of the interactions between NHS Smile4life staff and Third Sector staff, an essential component of Smile4life delivery that was previously unknown and under-explored in the literature. Other interventions designed to tackle the oral health of people experiencing homelessness are predominantly focused on provision of dental treatment and less often explore non-clinical interventions or the roles of non-dental practitioners (19). As such, investigating the roles and interactions between practitioners (both Smile4life and Third Sector) allowed for greater understanding of how this influenced implementation.

Following the focus groups, additional research has subsequently been conducted, exploring organisational factors, the effect of policy, and variation in, and influences on, the delivery of the Smile4life intervention (20). The critical reflection and learning generated from this study evaluating the implementation of Smile4life has also gone on to inform a follow up co-design project to produce the second Smile4life Guide for Trainers, with participation from people with lived experience and practitioners who use the guide to inform how the deliver the intervention (21).

### 4.3 Limitations

In all of the focus groups in this research, there were less than six participants and in once instance only two participants; less than the numbers usually recommended in the literature (6–8). However, in all three instances, everyone who was involved with Smile4life in each participating NHS Board took part. Fortunately, at no point did the discussion dry up until the Smile4life practitioners had answered all the questions, and all voices were heard, depending on participants' level of involvement with Smile4life.

Additionally, Kitzinger noted that while there are benefits to conducting focus groups with participants that already know each other, group norms can emerge that makes it difficult for participants to express disagreement or conflicting opinions (7). In the Smile4life focus groups, there was a sense that because the participants knew each other and worked together, they were supportive of each other, and comfortable to express other points of view. However, there were no significant disagreements, perhaps because of group norms or because the Smile4life practitioners genuinely agreed with each other.

In his work on boundary spanners, Williams created a job description for boundary spanners, factoring in their skills, qualifications, experience and their personal characteristics (17). Based on the findings from the focus groups, supported by the observation study, we can conclude that Smile4life practitioners do largely fit this description, at least in two of the three NHS Boards who took part. However, future research could perhaps examine this in more depth and compare Smile4life practitioners to this description more formally, or the job description could be used to identify practitioners that are particularly well suited to working on Smile4life.

Finally, it should be noted that this work took place prior to the COVID-19 pandemic and the current cost of living crisis affecting the UK, which have both posed significant challenges for vulnerable/excluded groups, including those experiencing homelessness, and also for practitioners tasked with implementing interventions such as Smile4life (22–24). The results should be considered with this in mind.

## 5 Conclusion

In conclusion, the focus groups with Smile4life practitioners revealed that the Smile4life intervention had provided practitioners with the capability, opportunity and motivation to engage with Third Sector services and service users. These factors varied depending on the circumstances of each NHS Board and the individual Smile4life practitioners' personal attributes and working experiences. Analyzing the focus groups using a framework based on the COM-B model allowed several factors to emerge that acted as barriers for the Smile4life practitioners. The most significant of these was the issue of poor relationships between the Smile4life practitioners and the Third Sector staff, reinforcing the findings from the earlier observation study. Additional barriers included unavailable resources, dental anxiety and negative past experiences leading to low motivation to make future attempts at engagement.

Further examination of the focus group discussions suggested that the Smile4life practitioners are those who, by necessity and their strong motivation to make a difference, must operate across fields or sectors, to achieve their goal and benefit service users. Smile4life practitioners demonstrate elements of the four aspects of boundary spanning, and their personalities and skills also point to practitioners who are well suited to the role of boundary spanning, something that had been noted during the previous observation study and had also emerged during the focus groups. Their boundary spanning skills go hand-in-hand with their capability to engage, as well as encouraging them to make their own opportunities, or take advantage of existing ones. Lastly, it is likely that Smile4life practitioners’ motivation for engagement was what allowed them to span boundaries, as it allowed them to “*go the extra mile*” in their Smile4life work and overcome risks to engage with service users. This suggests that the Smile4life intervention had influenced the engagement behaviours of practitioners, enhancing their capability, opportunity and motivation and facilitating boundary spanning.

## Data Availability

The datasets presented in this article are not readily available because of restrictions from the ethical committee. Please contact the corresponding author for more information. Requests to access the datasets should be directed to laura.beaton@nhs.scot.

## Footnotes

^1^For the purposes of this work, “Third Sector” refers to voluntary organisations, charities, community groups or social enterprises, which operate outside of, but alongside, the public or private sectors.

## References

[1] Beaton L Anderson I Humphris G Rodriguez A Freeman R. Implementing an oral health intervention for people experiencing homelessness in Scotland: a participant observation study. Dent J. (2018) 6:1–14. 10.3390/dj6040068 PMC6313649 30513716

[2] Freeman R Doughty J Macdonald ME Muirhead V. Inclusion oral health: advancing a theoretical framework for policy, research and practice. Community Dent Oral Epidemiol. (2020) 48:1–6. 10.1111/cdoe.12500 31625202

[3] Freeman R Coles E Watt C Edwards M Jones C. Smile4life guide for trainers: Better oral care for homeless people. Edinburgh: NHS Health Scotland (2012).

[4] FEANTSA. *European typology of homelessness and housing exclusion*. Available at: https://www.feantsa.org/download/en-16822651433655843804.pdf (Accessed May 09, 2023).

[5] Beaton L Freeman R. Oral health promotion and homelessness: a theory-based approach to understanding processes of implementation and adoption. Health Educ J. (2016) 75:184–97. 10.1177/0017896915571144

[6] Slattery P Saeri AK Bragge P. Research co-design in health: a rapid overview of reviews. Health Res Policy Syst*.* (2020) 18:17. 10.1186/s12961-020-0528-9 32046728 PMC7014755

[7] Kitzinger J. Qualitative research: introducing focus groups. BMJ (1995) 311:299. 10.1136/bmj.311.7000.299 7633241 PMC2550365

[8] Bloor M Frankland J Thomas M Robson K. Focus groups in social research. London: Sage Publications Ltd. (2001).

[9] Cohen D Crabtree B, Focus Groups. *Qualitative research guidelines project*. (2006). Available at: http://www.qualres.org/HomeFocu-3647.html (Accessed March 08, 2023).

[10] Barbour R. Doing focus groups. London: Sage Publications Ltd. (2007).

[11] Morgan DL Krueger RA. When to use focus groups and why. In: Morgan DL, editors. Successful focus groups: Advancing the state of the art. California: Sage Publications Inc. (1993). p. 3–19.

[12] Krueger RA Casey MA. Focus groups: A practical guide for applied research. 3rd ed. California: Sage Publications Inc. (2000).

[13] Srivastava A Thomson SB. Framework analysis: a qualitative methodology for applied policy research. J Manag Gov. (2009) 4:72–9. https://ssrn.com/abstract=2760705

[14] Michie S Atkins L West R. The behaviour change wheel: A guide to designing interventions. Great Britain: Silverback Publishing (2014).

[15] Scottish Executive. An action plan for improving oral health and modernising NHS dental services. Edinburgh: Scottish Executive (2005).

[16] Scottish Government. National oral health improvement strategy for priority groups: Frail older people, people with special care needs and those who are homeless. Edinburgh: Scottish Government (2012). https://www.feantsaresearch.org/public/user/Observatory/2022/EJH_16-2/EJH_16-2_TP1.pdf

[17] Williams P. Collaboration in public policy and practice. Perspectives on boundary spanners. Bristol: The Policy Press (2012).

[18] Williams P. The life and times of the boundary spanner. J Integr Care. (2011) 19:26–33. 10.1108/14769011111148140

[19] Beaton L Humphris G Rodriguez A Freeman R. Community-based oral health interventions for people experiencing homelessness: a scoping review. Community Dent Health*.* (2020) 37:1–11. 10.1922/CDH_00014Beaton11 32212437

[20] Beaton L Humphris G Rodriguez A Freeman R. Implementing the Smile4life intervention for people experiencing homelessness: a path analytical evaluation. BMC Oral Health*.* (2021) 21:383. 10.1186/s12903-021-01747-1 34353301 PMC8340521

[21] Rodriguez A Biazus-Dalcin C Marshall J Gorman R. Smile4life: A co-designed educational and training resource guide. Edinburgh: NHS Education for Scotland (2022).

[22] Broadbent P Thomson R Kopasker D McCartney G Meier P Richiardi M The public health implications of the cost-of-living crisis: outlining mechanisms and modelling consequences. Lancet Reg Health Eur. (2023) 27:100585. 10.1016/j.lanepe.2023.100585 37035237 PMC10068020

[23] Snell C Pleace N. The energy crisis and the homelessness crisis: emergent agendas and concerns. Eur J Homeless. (2022) 16:179–97.

[24] Watts B Bramley G Fitzpatrick S Pawson H Young G. The homelessness monitor: Scotland 2021. London: Crisis (2021).

