# Supplementary material for: Exploring the engagement behaviours of Smile4life practitioners: lessons from an evaluation of the national oral health improvement programme for people experiencing homelessness in Scotland
Source: Front Oral Health. 2024 Jan 4;4:1289348. doi: 10.3389/froh.2023.1289348 (PMC10794537; doi:10.3389/froh.2023.1289348)
Supplement: Supplementary file 1 [file Table1.docx]

**Supplementary File 1: Focus Group Questions**

| **Question** | **Prompt/Cue** |
| --- | --- |
| Welcome and Introductions | Who you are? Where you work? Job title? How long you have worked on Smile4life? |
| What are your experiences interacting with service providers? | While offering Smile4life training or when visiting to talk to service users |
| How do you approach services? |  |
| How would you describe these service providers? | Are they welcoming? Are they happy for you to speak to service users? Have they always been like that? Were they interested in receiving training in Smile4life? |
| Have you experienced any hostility or negativity from staff? |  |
| What are you most effective strategies to overcome this? |  |
| What are your perceptions about people experiencing homelessness? |  |
| How does society perceive them? |  |
| What are your experiences interacting with service users? | Have they gone well/not well? If not, why not? |
| How do you motivate service users to engage with you? | Incentives?  Building rapport? |
| Are there any hostility/difficulties/problems? |  |
| In general, what (other) barriers have you experienced while implementing Smile4life? | Not service user/service provider specific. e.g. not enough time, priorities |
| Tell me about a difficult situation that you have experienced?  How did you deal with this? | Not service user/service provider specific. What did you do to improve the situation? |
| Think back to when you began working on Smile4life, what were your expectations about working with people who were experiencing homelessness? |  |
| Were you concerned or apprehensive about any aspects of this work? |  |
| Comparing then with now, is working on Smile4life like what you expected? |  |
| Has anything changed between then and now? |  |
| Tell me about your experiences of the risks involved in your work with Smile4life? | e.g. feeling afraid, unsafe, challenges working with those experiencing homelessness |
| How do you feel about this element of your Smile4life work? |  |
| How do you deal with this risk? | e.g. keeping door open, being near reception |
| What skills do you think a practitioner needs, to work with people experiencing homelessness on Smile4life? | e.g. communication, tolerance |
| What helps you when you are working on Smile4life and interacting with people experiencing homelessness? | e.g. to make a difference, to achieve a positive outcome |
| Thinking about the Dental Action Plan and the Priority Groups Strategy, how have they affected your work? | e.g. Would you be doing this work even if these policies did not exist? |
| Are there any parts of your work that you don’t enjoy? |  |
| Are there any parts of your work that you particularly enjoy? |  |
| Is there anything else about your Smile4life work that you want to tell me about? |  |
