# Supplementary material for: Exploring the engagement behaviours of Smile4life practitioners: lessons from an evaluation of the national oral health improvement programme for people experiencing homelessness in Scotland
Source: Front Oral Health. 2024 Jan 4;4:1289348. doi: 10.3389/froh.2023.1289348 (PMC10794537; doi:10.3389/froh.2023.1289348)
Supplement: Supplementary file 2 [file Table2.docx]

Supplementary File 2: Thematic framework used to explore focus group data

| **Code** | **Subcategory** | **Smile4life Example** |
| --- | --- | --- |
| Capability | Physical | Are there sufficient staff and resources for practitioners to engage with Third Sector homelessness services and service users? |
|  | Psychological | Do practitioners have the skills needed to engage with Third Sector homelessness services and service users? |
| Opportunity | Physical | Do practitioners have access to the necessary services or service users? |
|  | Social | Is there a strong working relationship with the Third Sector? |
| Motivation | Automatic | Are practitioners influenced by their expectations regarding homelessness while delivering Smile4life and engaging with Third Sector homelessness services and service users? |
|  | Reflective | Do practitioners believe they are capable of engaging with Third Sector homelessness services and service users? |
